# Role of circular RNAs in regulating toxicity induced by cancer therapies

**DOI:** 10.1016/j.gendis.2025.101982

**Published:** 2025-12-11

**Authors:** Jiawen Xian, Javeria Qadir, Burton B. Yang, Ting Ye

**Affiliations:** a Department of Laboratory Medicine, The Affiliated Hospital of Southwest Medical University, Luzhou, Sichuan 646000, China; b Sunnybrook Research Institute, Sunnybrook Health Sciences Centre, Toronto, ON M4N 3M5, Canada; c Department of Laboratory Medicine and Pathobiology, University of Toronto, Toronto, ON M5S 1A1, Canada; d Department of Biosciences, COMSATS University Islamabad, Islamabad 444000, Pakistan

**Keywords:** Chemotherapy, circRNAs, Oncotherapy, Prognosis, Radiotherapy, Toxicity

## Abstract

Owing to transformative improvements in diagnosis and treatment, survival rates for cancer patients have improved significantly across the globe. However, toxicity induced by oncotherapy remains a major concern and markedly affects disease prognosis. In recent years, research on the association between circular RNAs (circRNAs) and oncotherapy-induced toxicity has received extensive attention. CircRNAs are a class of single-stranded closed-loop molecules that play a regulatory role in the occurrence and development of tumors. An integral role of circRNAs in the development of cancer treatment-induced toxicity, as well as in pathological processes such as oxidative damage, mitochondrial damage, apoptosis, dysregulation of calcium homeostasis, and dysregulation of vascular homeostasis has been deciphered. With regards to chemotherapy, radiotherapy, and immunotherapy for cancer treatment, circRNAs play crucial functions in modulating the effects of oncotherapy-induced toxicity. The current review focuses on the mechanisms by which circRNAs function in regulating cancer treatment-induced toxicity, which leads to apoptosis, mitochondrial damage, oxidative stress, DNA damage, and fibrosis. In addition, this review summarizes the potential circRNA biomarkers, treatment strategies and future challenges, which may help translate circRNA research into clinical practice for early detection and improvement of cancer treatment-induced toxicity in the future.

## Introduction

In recent decades, survival rates for cancer patients have increased significantly across the globe due to improvements in diagnosis and treatment.^1^ Currently, cancer treatment typically employs approaches that include surgery, chemotherapy, radiotherapy, targeted therapy, immunotherapy, and/or hormonal therapy. However, various side effects are associated with these strategies. For instance, chemotherapy lacks specificity and can kill tumor cells as well as normal cells/tissues.^2^ Radiotherapy can also cause varying degrees of damage to normal tissues.^3^ These side effects are considered the cause of death in cancer survivors and even exceed the mortality associated with tumor recurrence, affecting the short- and long-term prognosis of such patients.^4^ Currently, researchers have analyzed clinical specimens,^5^ carried out bioinformatics analysis,^6^ animal and cellular modeling,^7^ and several other methods to reveal the mechanisms by which various therapeutic modalities cause adverse effects on different organs. Chemical-induced cardiotoxicity,^8^ acute kidney injury,^7^ peripheral neuropathy,^9^ radiation-induced acute radiation syndrome of the gastrointestinal tract,^10^ acute esophagitis,^11^ and lung injury^12^ have been reported. These side effects, in the short and long term, can result in poor survival outcomes, and there is currently no effective method for their prevention.^13^^,^^14^ Such toxic effects can potentially impede the expected therapeutic outcomes, thus disrupting the course of cancer treatment and adversely affecting quality of life.^15^ The toxicity of anti-cancer therapy may be a major underlying factor in the development of more effective diagnostic interventions. Therefore, a deeper understanding of the molecular mechanisms that regulate cancer treatment-induced toxicity is needed to develop more effective diagnostic and therapeutic approaches. With the emergence of high-throughput RNA sequencing (RNA-seq), numerous circRNAs have been identified and characterized in humans as well as other eukaryotes.^16^^,^^17^ CircRNAs are a class of single-stranded, closed-loop molecules without 5′ and 3′ ends and a poly-A tail, which renders them resistant to exonucleases, thus making them more stable than their linear counterparts.^18^ Numerous studies on the diverse cellular functions of circRNAs have revealed essential functional implications in the process of tumorigenesis, proliferation, invasion, metastasis, stem cell regulation, and radio-resistance, endorsing their utility as putative biomarkers and therapeutic targets for cancer management.^19^^,^^20^ Previously, many studies focused on circRNA-mediated regulation of tumorigenesis and progression.^19^^,^^21^^,^^22^ However, with the inception of more comprehensive studies, an integral role of circRNAs in the development of cancer treatment-induced toxicity (mainly cardiotoxicity),^5^ as well as in the pathological processes such as oxidative damage, mitochondrial damage, apoptosis, dysregulation of calcium homeostasis, and dysregulation of vascular homeostasis, has been deciphered.^23^ Pertinently, the use of the chemotherapeutic agent doxorubicin has been observed to upregulate the expression of circ_0001312, which promotes cardiomyocyte apoptosis, inflammation, and oxidative stress through the miR-409-3p/HMGB1 axis.^8^ On the basis of these observations, altered circRNA expression may serve as a useful measure for timely detection of toxicity associated with anti-cancer therapies, which is critical for improving cancer prognosis. In addition, circRNAs have the potential to be employed as therapeutic targets to mitigate cancer treatment-induced cardiotoxicity.^5^^,^^24^ Deciphering the functional mechanisms of circRNAs in modulating cancer therapy-induced toxicity may contribute to the development of new assays and effective therapeutic measures. Hence, this review aims to provide up-to-date information on the toxicity associated with contemporary anti-cancer therapies. Moreover, we summarize the generation and function of circRNAs and their possible mechanisms of action in cancer therapy-induced toxicity and discuss the clinical potential of these circRNAs as biomarkers and therapeutic targets. Finally, we discuss the needs of advanced research in this field and the challenges that need to be addressed in an attempt to move it towards clinical practice.

## General outlook on circRNAs

### CircRNA biogenesis and characteristics

**Figure 1:**
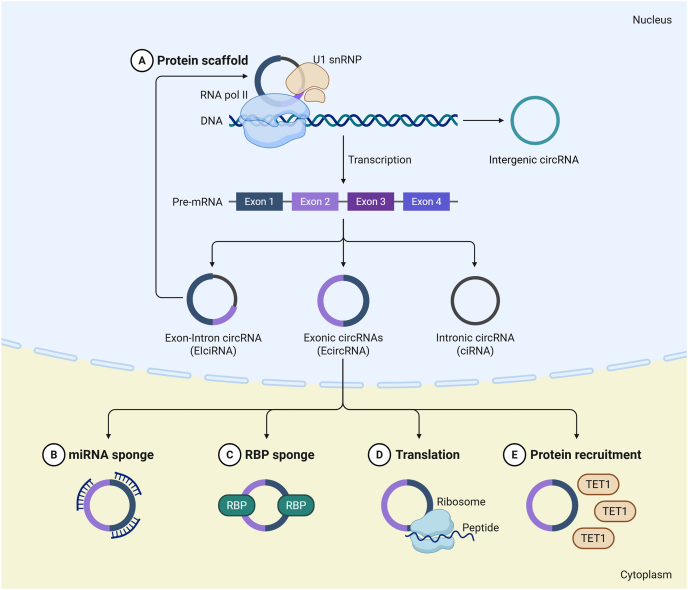
Biogenesis and functional mechanisms of circRNAs. circRNAs are produced via biogenesis models driven by lariat-mediated back-splicing (BS), RNA binding protein (RBP), or intronic complementary sequences (ICS), resulting in the formation of either exonic (EcircRNA), intronic (ciRNA) or exon‒intron (EIciRNA) circRNAs. Mechanistically, circRNAs perform pathophysiological functions via acting as **(A)** protein scaffolds, **(B)** miRNA sponges, **(C)** RNA binding protein (RBP) sponges, **(D)** translation into a functional peptide, and **(E)** protein recruitment.

A circRNA is a single-stranded, covalently closed endogenous biomolecule that is produced from pre-mRNA as a consequence of back-splicing, in which the downstream 3′ splice site is linked across one or more exons to the upstream 5′ splice site.^25^ The process of back-splicing for circRNA generation can occur at the transcription level as well as post-transcriptionally, and is largely mediated through a canonical spliceosome-based mechanism.^26^ Moreover, back-splicing is distinctively influenced by specific cis- and trans-acting elements, which strictly regulating the formation of circRNAs.^27^ Interestingly, alternative back-splicing and alternative splicing site selection at a specific gene locus can occur during circRNA synthesis, culminating in the generation of more than one RNA.^28^ CircRNAs can typically be categorized as exonic circRNAs, circular intronic RNAs, exon‒intron circRNAs or mitochondria‒encoded circRNAs (mecciRNAs),^29^ as depicted in Figure 1. Most endogenously expressed circRNAs in humans are composed of either two or three exons. Various studies have described circRNAs as erroneous products of mRNA splicing with no evident function.^30^ However, some studies have also reported high expression of circRNAs compared to their linear cognate mRNAs, highlighting the fact that circRNA expression is not dependent upon its corresponding linear mRNA.^31^ CircRNAs do not possess the 5′-cap structure or 3′-poly(A) tail of linear RNA molecules. Like linear RNA molecules, circRNA molecules rely on the 3′-5′ phosphodiester bond for the linkage between nucleotides.^32^ CircRNAs are produced inefficiently at the transcriptional level but can gradually accumulate to high levels of expression through continuous biogenesis and decreased degradation.^33^ Notably, most circRNAs are not only resistant to degradation caused by linear RNA decay mechanisms but also have longer half-lives and higher stability compared to the homologous linear RNAs.^34^ In addition, the expression of numerous circRNAs is cell-specific, tissue-specific and disease-specific and shows remarkable potential in tumor pathogenesis.^35^

### Functional mechanisms of circRNAs

Since 2013, a growing body of research has revealed the mechanisms underlying circRNA function.^36^ Specifically, these mechanisms; include (i) acting as microRNA (miRNA) sponges, (ii) regulating splicing and transcription, (iii) being translation into proteins, and (iv) functioning through interactions with proteins,^37^, ^38^, ^39^ as illustrated in Figure 1. Predominantly, one circRNA may display different mechanisms to perform its biological functions depending on the specific type of circRNA and the functional environment.^40^ Most circRNAs are exon-derived, thus participating in the post-transcriptional regulation in the cytoplasm, mainly via sponging miRNAs.^41^ These circRNAs, called competing endogenous RNAs (ceRNAs), can silence miRNAs through complementary base pairing, thus inhibiting miRNA-mediated mRNA degradation and “rescuing” target gene expression.^42^ In contrast, intronic circRNAs usually cis-regulate gene transcription in the nucleus. This process involves interactions with nuclear proteins such as RNA polymerase II (Pol II) or U1 small nuclear ribonucleoproteins (snRNPs).^43^ Moreover, circRNAs can bind to proteins for further biological functions.^44^ In recent years, the discovery of internal ribosome entry sites (IRESs) in circRNAs that can bind to ribosomes or open reading frames (ORFs) has opened avenues for studying and elucidating the potential of circRNAs.^45^

### CircRNAs and chemotherapy-induced toxicity

**Figure 2:**
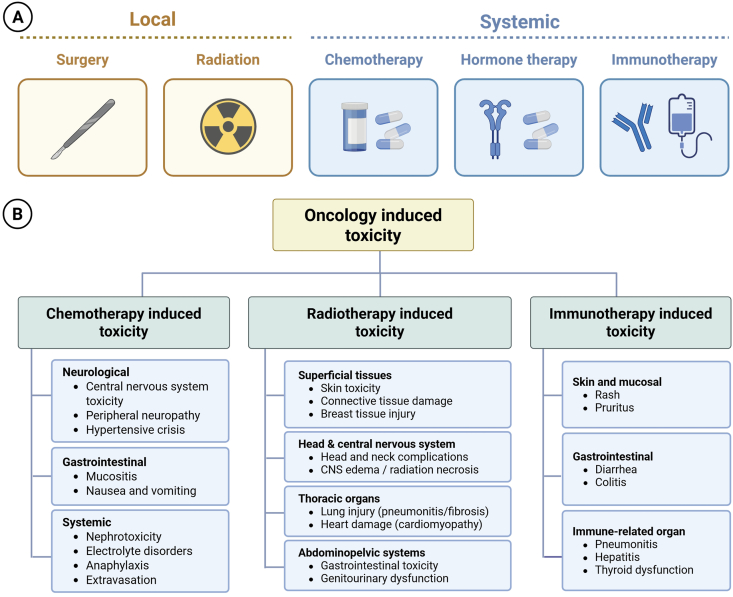
Onco-therapy-induced toxicity. **(A)** Illustration of various modes of cancer therapy, whereby local methods include (i) surgery and (ii) radiation exposure, and systemic methods include (i) chemotherapy, (ii) hormonal therapy and (iii) immunotherapy, and **(B)** a list of adverse effects and organ damage associated with the most commonly employed methods of oncotherapy.

The therapeutic management of cancer generally employs approaches that include surgery, chemotherapy, radiotherapy, targeted therapy, immunotherapy, and/or hormonal therapy.^46^ The relevant local and systemic methods of cancer therapy are shown in Figure 2. However, cancer treatment is often associated with a wide range of adverse effects, referred to as “oncotherapy-induced toxicity”. Unfortunately, due to these adverse effects, cancer survivors usually eventually die, as evidenced in patients with breast, prostate or brain cancer.^47^

Among the various methods used in cancer therapy, chemotherapy and radiotherapy have been most extensively studied for their role in inducing adverse or toxic effects in patients diagnosed with any form of cancer, thus influencing the short- and long-term prognosis.^48^^,^^49^ The toxic effects induced by chemotherapeutic agents include (i) cardiotoxicity, (ii) nephrotoxicity, and (iii) neurotoxicity. The subsequent section discusses these effects and the circRNAs potentially implicated in modulating such effects in various cancer type, as listed in Table 1.

**Table 1:** List of circRNAs associated with oncotherapy-induced toxicity.

Oncotherapy	Organ system affected	CircRNA symbol/ID	Expression status	Potential mechanism	Functional implication	Reference
Chemotherapy	Cardiotoxicity	circ_0001312	High	miR-409-3p sponge	HMGB1 overexpression promoting cardiotoxicity	8
		circ-ZNF609	High	Regulates m^6^A demethylase FTO	DOX-induced cardiomyocyte cell death	56
		circ-LTBP1	High	Sponging miR-107 and elevating ADCY1.	DOX-induced intracellular toxicity in cardiomyocytes	57
		circ-INSR	High	Interacts with the single-stranded DNA-binding protein SSBP1	Cardioprotective role	62
		circITCH	Low	Endogenous sponge for miR-330-5p	Upregulating SIRT6, Survivin, and SERCA2a to alleviate DOX-induced cardiomyocyte injury and dysfunction	5
		circ-30741	High	miR-21 sponge	Drug-mediated cardiomyocyte injury and fibrosis	69
	Nephrotoxicity	circ-0114427	High	miR-494 sponge	Early inflammatory progression of CP-AKI	7
		circRNA_3907	High	miR-185-3p sponge	PTPRN transmembrane regulation	76
	Neurotoxicity	mmu_circ_0009357	High	miRNA regulation	PTX-induced CIPN	83
		mmu_circ_0013069				
		mmu_circ_0006031				
		mmu_circ_0001817				
Radiotherapy	Gastrointestinal toxicity	circRNA_2909	High	Sponging miRNAs	Increase HIF1A and NOS2 in the HIF-1 pathway, thus, producing radioprotective effects in the gastrointestinal tract	92
		circRNA_0323				
	Pulmonary toxicity	circRNA3340	High	miR-146a-5p sponge	Immunomodulatory effect via CD4 regulation	6
		circRNA544	High	miR-188-3p sponge	Immunomodulatory effect via IL2Ra regulation	
		circRNA439	High	miR-702-3p sponge	Immunomodulatory effect via IL12a regulation	
	Cardiotoxicity	circFOXO3	High	Regulating Bax, caspase 3 and 7, and Bcl-2 expression	Protects cardiomyocytes from radiation-induced cardiotoxicity by reducing DNA damage and apoptosis	24

Notes: CIPN, chemotherapy-induced peripheral neuropathy; CP-AKI, cisplatin-induced acute kidney injury; DOX, doxorubicin.

### Chemotherapy-induced cardiotoxicity

Survival rates for cancer patients have increased significantly with the development of chemotherapeutic drugs.^50^ However, many chemotherapeutic agents cause adverse effects, with cardiovascular toxicity being one of the most common and life-threatening adverse effects.^1^ Anthracyclines are a class of chemotherapeutic agents used to treat malignant tumors such as lymphoma, sarcoma, and breast cancer.^51^ The most commonly used anthracycline is doxorubicin (DOX). However, up to a quarter of patients experience DOX-induced cardiotoxicity, which limits its clinical application.^52^ Mechanistically, DOX can produce its antitumor effect by targeting DNA in one of two ways. On the one hand, DOX enters the cell and inserts itself between the DNA base pairs, thus blocking DNA replication and RNA transcription.^53^ On the other hand, DOX targets topoisomerases, enzymes that cleave and unfold DNA to allow DNA replication. DOX stabilizes the conformation of the cleaved DNA strand at the site where it binds to topoisomerases, which prevent DNA double-strand from resealing and can ultimately lead to cellular senescence or apoptosis.^54^ The primary mechanism underlying DOX-induced toxicity is mediated by oxidative stress, which may occur as a consequence of mitochondrial dysfunction, inflammation, DNA damage, disruption of protein degradation pathways, signaling through cell death pathways, and damage to cardiac progenitor cells.^55^ CircRNAs can both promote and ameliorate DOX-induced cardiotoxicity.^4^ Some circRNAs can promote cardiotoxicity; for example, DOX has been reported to promote circ_0001312 expression, and knockdown of circ_0001312 reverses DOX-induced cardiomyocyte injury. Mechanistically, DOX upregulated circ_0001312, which competitively binds to miR-409-3p, and reduces the inhibitory effect of miR-409-3p on HMGB1, thereby increasing the expression of HMGB1. In addition, miR-409-3p attenuated DOX-induced apoptosis, inflammation, and oxidative stress in cardiomyocytes, and these effects were counteracted by HMGB1 overexpression. Thus, the inhibition of circ_0001312 reversed the DOX-mediated cytotoxic effects on cardiomyocytes via the miR-409-3p/HMGB1 axis.^8^ Similarly, circ-ZNF609 is a specialized circRNA with RNA m6A modification that regulates the m6A demethylase FTO. circ-ZNF609 expression was significantly increased in the heart after DOX treatment. Knockdown of circ-ZNF609 expression *in vivo* attenuated DOX-induced cardiotoxicity by reducing pathological remodeling (including apoptosis and fibrosis) while preserving cardiac function. Conversely, overexpression of circ-ZNF609 promoted DOX-induced cardiomyocyte death. Moreover, circ-ZNF609 stability was reduced by a decrease in RNA m^6^A modification, and the m^6^A demethylase FTO, a downstream effector of circ-ZNF609, was negatively regulated and elevated, which led to a further decrease in RNA m^6^A modification, as well as increased reactive oxygen species (ROS) production, iron death, and mitochondrial iron overload, leading to cardiotoxicity. Therefore, circ-ZNF609 has potential implications for promoting DOX-induced cardiotoxicity.^56^ In addition, circ-LTBP1 is involved in DOX-induced intracellular toxicity in cardiomyocytes through miR-107/ADCY1 signaling.^57^ In AC16 cells, DOX induced the upregulation of circ-LTBP1, which up-regulated ADCY1 expression via sponge adsorption of miR-107, thus inhibiting cell proliferation and promoting inflammation, apoptosis, and oxidative stress. In contrast, inhibition of circ-LTBP1 or up-regulation of miR-107 to down-regulate ADCY1 produced the opposite effect. Therefore, circ-LTBP1 was found to enhance DOX-induced effects on proliferation inhibition, inflammation, apoptosis and oxidative stress in AC16 cells by competitively sponging miR-107 and increasing ADCY1. Likewise, circFOXO3 has been reported to promote cardiac senescence by interacting with ID-1, E2F1, FAK, and HIF1α, causing their cytoplasmic retention, thus withholding their anti-stress and anti-senescent effects.^44^ Similarly, some circRNAs may ameliorate chemotherapy-induced cardiotoxicity. Qki5, an RNA-binding protein, is the most abundant Quaking family member in the heart and has been reported to regulate circRNA biogenesis and inhibit cardiomyocyte apoptosis during the epithelial–mesenchymal transition (EMT) in an ischemia‒reperfusion model.^58^^,^^59^ DOX-induced down-regulation of Qki5 inhibited circRNAs derived from Ttn, Fhod3, and Strn3 in mouse cardiomyocytes, thereby increasing the rate of DOX-induced apoptosis. Further *in vivo* studies suggested that moderate overexpression of Qki5 may be necessary for its protective effect against DOX-induced cardiotoxicity in mice.^60^ Similarly, circ-Amotl1 has been revealed to physically interact with PDK1 and AKT1, mediating AKT phosphorylation and facilitating the nuclear translocation of pAKT, thus reducing apoptosis and producing a cardio-protective effect.^61^ Another study revealed that circ-INSR was downregulated in rodents and patients with chemotherapy-induced cardiotoxicity, resulting in cardiomyocyte death, cardiac dysfunction, and mitochondrial damage. In contrast, overexpression of circ-INSR was observed to play a cardioprotective role in a DOX-induced cardiotoxicity mouse model.^59^ Breast cancer type 1 susceptibility protein (BRCA1) is a regulator of circ-INSR expression. circ-INSR physically interacts with the single-stranded DNA-binding protein SSBP1 to mediate its cardioprotective effects in response to adriamycin.^62^ In addition, circITCH has been reported to be downregulated in DOX-treated human induced pluripotent stem cell hiPSC-derived cardiomyocytes (hiPSC-CMs). Similarly, it was downregulated in autopsy specimens from cancer patients suffering from DOX-induced cardiomyopathy. Mechanistically, circITCH acts by acting as an endogenous sponge for miR-330-5p. SIRT6, Survivin, and SERCA2a are upregulated by the circITCH/miR-330-5p axis to alleviate DOX-induced cardiomyocyte injury and dysfunction. Moreover, circITCH is a novel therapeutic hotspot for DOX-induced cardiotoxicity.^5^

In addition to DOX, another chemical agent, arsenic trioxide (As2O3, ATO), has limited use in the clinic due to its cardiotoxicity.^63^ Originally used in traditional Chinese medicine, ATO has gradually become a first-line antitumor drug for the treatment of acute promyelocytic leukemia.^64^ However, ATO use can lead to adverse cardiac effects, which are characterized by long QT syndrome, tachycardia, and sudden cardiac death.^63^ Previous studies have shown that ATO induces oxidative stress-mediated cardiotoxicity by causing cardiomyocyte apoptosis and redox state imbalance either by activating the caspase-3 pathway or by mediating changes in internal mitochondrial membrane potential (MMP).^65^

In addition, the imbalance of trace elements caused by ATO may lead to altered mitochondrial dynamics, which induce apoptosis and limit cellular metabolism. Jiang et al^66^ elucidated the molecular mechanism of ATO-induced cardiotoxicity by analyzing the transcriptome and constructing a circRNA‒lncRNA network in the myocardium of ATO-treated mice. Among the 94 differentially expressed circRNAs in the myocardium, 49 were upregulated, whereas 45 were downregulated. The investigators subsequently constructed a circRNA‒miRNA‒mRNA network containing 9 circRNAs, 5 miRNAs, and 8 mRNAs. The aberrantly expressed circRNAs may be involved in ATO-induced cardiotoxicity by interacting with protein-coding genes and/or regulating miRNAs to exert their biological functions. This study provides new strategies for the prevention and treatment of ATO-induced cardiotoxicity.

Research on the functional involvement of circRNAs in chemotherapy-induced cardiotoxicity is still in its infancy. However, growing evidence indicates that circRNAs are associated with various cardiovascular diseases. For instance, circNCX1, transcribed from the sodium/calcium exchange protein 1 gene, plays a regulatory role in myocardial infarction.^67^ Furthermore, circRNA HRCR is involved in the regulation of cardiac hypertrophy.^68^ Additionally, circRNA30741 targets miR-21,^69^ while miR-21 is regulated by transforming growth factor β1 (TGF-β1) and is involved in drug-mediated cardiomyocyte injury and fibrosis. All of these above-mentioned circRNAs may play a role in chemotherapy-induced cardiotoxicity and are expected to be used as markers of cardiotoxicity in the therapeutic management of cancer.

### Chemotherapy-induced nephrotoxicity

Acute kidney injury (AKI) is a complex disease characterized by a rapid decline in the glomerular filtration rate (GFR) and an increase in the serum creatinine level. AKI has multiple etiologies, such as renal ischemia, rhabdomyolysis and toxicity.^70^ The incidence of pharmacologic AKI is increasing every year due to the development of more aggressive treatment strategies. Renal tubular tissue is the main target of nephrotoxicity, and tubular cell death is the most common pathological change in AKI.^71^ Among the chemotherapeutic agents commonly employed for cancer treatment, cisplatin is a major cause of acute kidney injury. It is a platinum-based inorganic compound that crosslinks DNA, thereby inhibiting essential processes that include DNA replication and transcription.^72^ Rapidly proliferating cancer cells are particularly sensitive to cisplatin-induced DNA damage owing to a relatively high rate of DNA replication. Depending on the extent of DNA damage, cancer cells either repair or tolerate DNA damage or undergo apoptosis if the damage is extensive.^73^ Cisplatin-induced acute kidney injury (CP-AKI) is an adverse effect of cisplatin that can increase susceptibility to chronic kidney disease.^74^ Clinically, CP-AKI is characterized by elevated serum creatinine levels, decreased urine output, and impaired renal function.^75^ CP-AKI is associated with short- and long-term adverse survival outcomes, and no effective prevention or therapy is currently available.^13^

Cao et al^7^ established a CP-AKI mouse model and isolated renal tubular tissues. The circRNAs were extracted and analyzed for composition, distribution, and source genes, and screened for differentially expressed circRNAs associated with AKI. While searching for homologous genes between mouse and human, they identified circ-0114427 in human cells, and observed its significant upregulation in different CP-AKI cell models. Mechanistically, circ-0114427 acts by sponging miR-494 to regulate ATF3 expression and further affects the expression of the downstream cytokine IL-6. Thus, circ-0114427 regulates the early inflammatory progression of CP-AKI through the circ-0114427/miR-494/ATF3 pathway. In another study, Ding et al^76^ established a CP-AKI mouse model by intraperitoneal injection of cisplatin, screened for differentially expressed ncRNAs compared with the saline-injected group, explored the mechanism by which the ceRNA network plays a role, verified that circRNA_3907 was upregulated in the CP-AKI model, and confirmed the presence of the circRNA_3907/mmu-miR-185-3p/PTPRN network. PTPRN is a transmembrane protein that upregulates pancreatic β-cell transcription and proliferation.^77^ miR-185-3p improves renal function in mice with diabetic kidney disease (DKD).^78^ Based on these data, circRNA_3907 upregulation may promote CP-AKI through the mmu-miR-185-3p/Ptprn axis, thereby associating the pathway with DKD.

### Chemotherapy-induced neurotoxicity

Chemotherapy-induced peripheral neuropathy (CIPN) is a common and consequential long-term adverse effect of several first-line chemotherapeutic agents, including paclitaxel (PTX). PTX binds to microtubules in the cytoskeleton and enhances microtubule protein polymerization, hence leading to apoptosis.^79^ PTX-mediated neurotoxicity damages the dorsal horn of the spinal cord, resulting in sensory abnormalities and mechanical abnormalities associated with pain.^80^ Approximately 40% of cancer survivors may experience lifelong symptoms and incapacitation due to CIPN.^81^ CIPN can cause a significant financial burden on patients as well as the healthcare system.^82^ Unfortunately, there are no effective strategies to prevent or limit the occurrence of CIPN.^14^ Cao et al^9^ used PTX to construct a CIPN model, screened for differentially expressed circRNAs, and established a circRNA‒miRNA‒mRNA network comprising 15 circRNAs, 18 miRNAs, and 11 mRNAs, which further identified *Cdh1*, *Satb2*, *Fas*, *P2ry2*, and *Zfhx2* as hub genes, suggesting a critical role of circRNAs in chemotherapy-induced CIPN. In another study by Mao,^83^ a PTX-induced neuropathic pain model was constructed and subjected to RNA-Seq, and 16 differentially expressed circRNAs were screened, among which, compared with those in control mice, PTX-treated mmu_circ_0009357, mmu_circ_0013069, mmu_circ_0006031, and mmu_circ_0001817 were expressed at different levels, with 1.37-fold, 1.33-fold, 1.40-fold and 1.50-fold higher expression levels, respectively. These finding suggest that these four circRNAs may be related to PTX-induced CIPN.

### CircRNAs and radiotherapy-induced toxicity

Radiation is a physical agent used to destroy cancer cells. Radiation therapy is used to treat more than half of all cancer patients and plays a key role in the therapeutic management of approximately 25% of all cancer types.^84^ The radiation used in radiotherapy is referred to as "ionizing radiation" because it forms ions and deposits energy in the cells of tissues it passes through. This deposited energy can either kill cancer cells or cause genetic changes that lead to cancer cell death.^85^ High-energy radiation damages cellular DNA, thereby blocking its ability to divide and proliferate further.^86^ While radiotherapy plays an important role in cancer treatment, it can be equally damaging to normal tissues surrounding cancerous tissues.^87^ Therefore, the goal of radiation therapy is to maximize the radiation dose to abnormal cancer cells while minimizing the exposure of normal cells, whereby normal cells can usually repair themselves much faster, thus maintaining their normal function.^85^ In general, cancer cells are not as effective as normal cells in repairing radiation-induced damage, resulting in varying degrees of cancer cell death.

### Radiotherapy-induced gastrointestinal (GI) toxicity

The gut is one of the most sensitive organs with respect to radiation-mediated toxicity.^88^ Radiation therapy for abdominal and pelvic tumors can lead to radiation-induced intestinal injury. Radiologic bone marrow injury can be mitigated by bone marrow transplantation, but there is no effective approach of preventing or curing radiation-induced intestinal injury.^89^ Radiation-induced intestinal toxicity is usually characterized by proliferative cell death and crypt apoptosis, inadequate replacement of the villous epithelium, disruption of the mucosal barrier, and marked inhibition of compensatory proliferation.^90^ The effects of toxicity on the intestinal tract are collectively referred to as ‘acute radiation syndrome of the gastrointestinal tract’.^10^ Its clinical manifestations include anorexia, vomiting, diarrhea, dehydration, and systemic infections, and in severe cases, infectious shock culminates in death. In Lu's study,^91^ a group of mice were compared with control mice for differentially expressed circRNAs in the jejunum 3.5 days after radiotherapy, the stage of the most severe intestinal injury, and 10 circRNAs were validated (chr18:35610871-35613502+, chr15:95864225-95894541+, chr3. 96041338-96042928-, chr5:64096979-64108263+, chr19:16705875-16710941-, chr5:134491893-134500149-, chr19:42562552-42564341+, chr5. 32640331-32664400+, chr3:72958113-72960367- and chr8:79343654-79372364-) for expression changes. Moreover, Yang et al^92^ reported that circRNA_2909 and circRNA_0323 promote the expression of HIF1A and NOS2 in the HIF-1 pathway by sponging miRNAs, which promotes epithelial integrity and reduces the intracellular levels of reactive oxygen species (ROS), hypoxia-inducible factor (HIF), and HIF1A and NOS2. Regarding, HIF has been shown to exhibit radioprotective effects in the gastrointestinal tract, which provides a new therapeutic strategy to alleviate radiation-induced gastrointestinal syndrome.

In the digestive system, in addition to the gastrointestinal tract, esophageal epithelial cells are extremely sensitive to ionizing radiation and are therefore also susceptible to damage caused by high-energy radiation during radiation therapy.^93^ Radiation-induced esophageal injury usually occurs in patients receiving radiation therapy for cervical, thoracic, or mediastinal cancer.^94^ Radiation acute esophagitis is the cause of radiation therapy suspension or failure.^11^ Ionizing radiation has also been reported to increase the risk of esophageal cancer in patients receiving radiotherapy for primary cancers of the head and neck, breast, and mediastinal regions.^95^, ^96^, ^97^ In a study by Luo et al^98^ circRNA expression profiles were compared between irradiated rats and normal controls via RNA-Seq analysis. Differentially expressed circRNAs are involved in cellular macromolecular metabolism, ion binding, enzyme binding, nucleotide binding, and the composition of cellular constituents, the most important of which is sphingolipid metabolism. Sphingolipids are membrane lipids that regulate lipid bilayer fluidity and substructural domain structure.^99^ Certain types of sphingolipids can function as biological effector molecules involved in various biological processes, including apoptosis, cell proliferation, cell migration, and inflammation.^100^ Thus, sphingolipids play crucial roles in the development and progression of cancer and may affect the efficacy of anti-cancer therapy.^101^ The expression of circRNAs associated with sphingolipid metabolism changed significantly during irradiation, suggesting that circRNAs may be responsible for radiation-induced damage in the esophagus through sphingolipid metabolism.

### Radiotherapy-induced pulmonary toxicity

Another radiotherapy-induced adverse effect is radiation-induced lung injury (RILI), which is characterized by acute pneumonitis and chronic fibrosis, both of which are potentially fatal and occur in approximately 10%–20% of patients receiving chest radiotherapy.^12^ RILI is a complex process involving a variety of molecular and cellular interactions that culminate in the proliferation, accumulation, and differentiation of large numbers of fibroblasts, leading to excessive extracellular matrix deposition and lung fibrosis.^102^ Li et al^6^ constructed a mouse model in which the mice were subjected to 12 Gy of chest irradiation, and lung tissues collected 48 h after irradiation were compared with normal lung tissues to screen for circRNAs with significantly different expression and perform gene ontology analysis of their target mRNAs. Among the 27 differentially expressed circRNAs, 10 were downregulated and 17 were upregulated in the irradiated group. These circRNAs are associated with Th1 and Th2 differentiation pathways. In conclusion, the authors tentatively confirmed the immunomodulatory role of the circRNA3340/miR-146a-5p/CD4, circRNA544/miR-188-3p/Il2ra and circRNA439/miR-702-3p/Il12a pathways in RILI.

### Radiotherapy-induced cardiotoxicity

**Figure 3:**
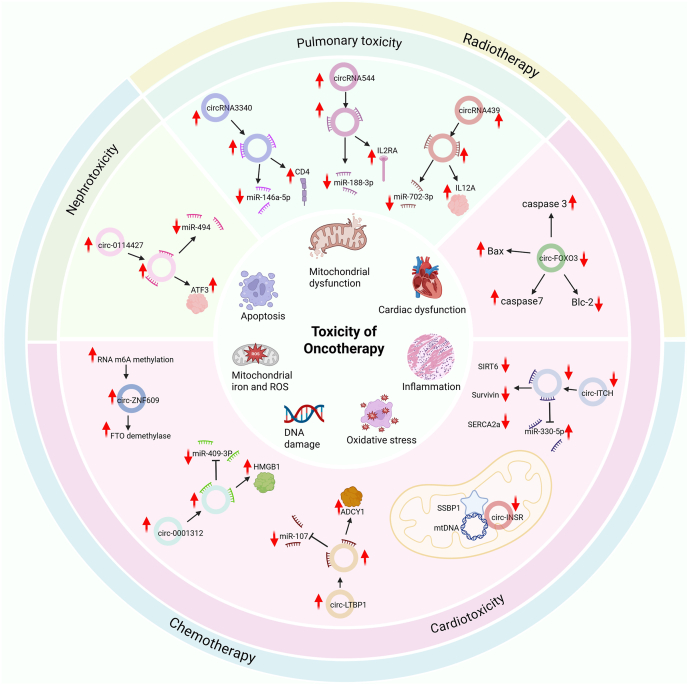
Adverse effects of oncotherapy-induced toxicity and the putative circRNAs implicated in their regulation in cancer. The outermost circle shows the mode of cancer therapy, chemotherapy (blue) and radiotherapy (skin); the middle circle represents the organ system affected by these therapies, cardiotoxicity (purple), nephrotoxicity (green), and pulmonary toxicity (blue); next, the circle represents the functional mechanisms of the circRNAs implicated in each of these cancer-induced toxicities; and the innermost circle encompasses the cellular processes affected (apoptosis, mitochondrial damage, cardiac dysfunction, inflammation oxidative stress, and DNA damage).

Unlike the aforementioned adverse effects of radiotherapy in the short term, which affect the GI tract and lungs, the specific cardiac manifestation of radiotherapy-induced toxicity is delayed cardiac damage.^48^ Over the past two decades, radiotherapy has been found to increase the risk associated with cardiac injury in cancer survivors.^103^ Similarly, knockdown of circFOXO3 in cardiomyocytes was associated with a significant increase in DNA damage and apoptosis after radiotherapy. Conversely, circFOXO3-overexpressing cells exhibited reduced rates of DNA damage and apoptosis. Moreover, circFOXO3 knockdown elevated Bax, caspase 3 and caspase 7 levels and decreased Bcl-2 expression, whereas circFOXO3 overexpression produced the opposite results. Additionally, circFOXO3 protects cardiomyocytes from radiation-induced cardiotoxicity by reducing DNA damage and apoptosis. Therefore, circFOXO3 may be a potential therapeutic target for radiation-induced cardiotoxicity in cancer patients.^24^ Figure 3 comprehensively depicts all the circRNAs that have been reported to modulate oncotherapy-induced toxicity in cancer patients, along with their putative mechanism of function.

### CircRNAs and immunotherapy-induced toxicity

Immunotherapy has revolutionized cancer treatment by harnessing the body's immune system to target and eliminate tumor cells. Immune checkpoint inhibitors (ICIs), such as anti-PD-1/PD-L1 and anti-CTLA-4 antibodies, have shown remarkable efficacy in multiple cancer types.^104^ However, their clinical success is often hampered by immune-related adverse events (irAEs), which can affect the skin, gastrointestinal tract, endocrine glands, liver, and other organ.^105^ These toxicities stem from aberrant or overactive immune responses and represent a major barrier to broader application. Recent studies suggest that circRNAs may regulate the onset and progression of immunotherapy-induced toxicity,^106^ although this area remains relatively unexplored.

Emerging evidence suggests the potential involvement of circRNAs in modulating immune cell function and inflammatory pathways, which may contribute to irAEs.^105^^,^^106^ For example, certain circRNAs have been found to regulate T-cell activation and differentiation, cytokine production, and antigen presentation, the key processes affected during immune checkpoint blockade.^107^, ^108^, ^109^ CircRNAs such as circHIPK3, circFOXO3, and circRNA_002178 have been implicated in immune signaling pathways, including the NF-κB, JAK/STAT, and PI3K/AKT pathways, which are also involved in the pathogenesis of immunotherapy-induced toxicity.^110^, ^111^, ^112^ Moreover, studies in autoimmune and inflammatory conditions that share mechanistic overlaps with irAEs have shown that dysregulated circRNA expression can exacerbate immune dysregulation.^109^ For instance, circRNAs that modulate the expression of IL-6, TNF-α, or IFN-γ may indirectly influence the severity of immunotherapy-associated inflammation.^113^^,^^114^ While direct links between circRNAs and specific irAEs are still being elucidated, the parallels with autoimmune biology provide a compelling rationale for deeper investigation. Functional studies and clinical validation are needed to determine whether targeting circRNAs can mitigate toxicity without compromising therapeutic efficacy.

With breakthroughs in the *in vitro* synthesis of circRNAs, artificial circRNAs have been engineered as a novel class of vaccines for disease treatment and prevention. Compared with the canonical linear mRNAs used in vaccines, circRNAs exhibit greater stability and lower immunogenicity.^115^ The cytotoxicity and side effects caused by mRNA vaccines are partly due to their high immunogenicity. Compared with modified mRNAs, which have somewhat modulated high immunogenicity, circRNAs exhibit lower immunogenicity and lower cytotoxicity in the absence of modification.^116^ However, circRNAs can still activate the innate immune system. Chen et al ^117^ were the first to demonstrate that transfection of engineered circRNA can stimulate the expression of several immune genes, with the most significant being retinoic acid-inducible gene-I (RIG-I). Future research on circular RNA vaccines for cancer treatment and prevention should also pay attention to treatment toxicity.

**Figure 4:**
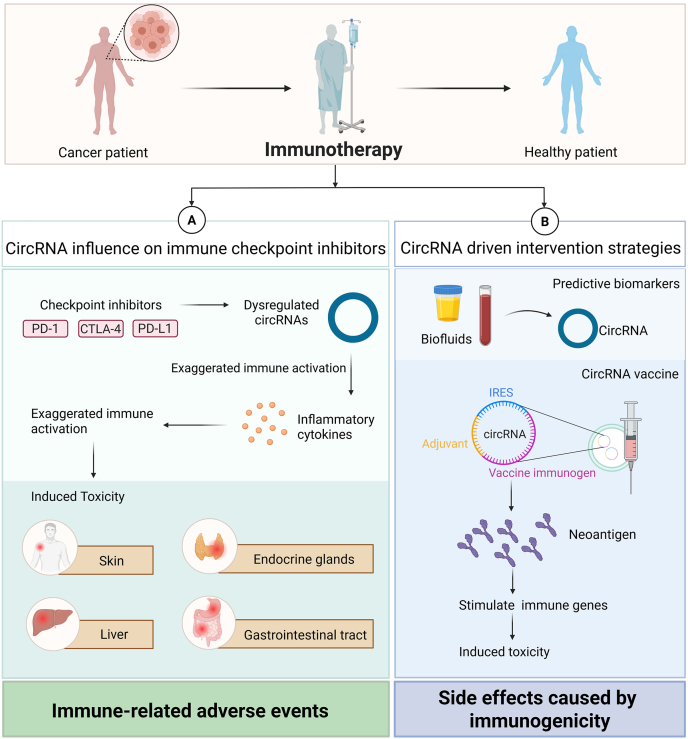
Regulatory roles of circRNAs in cancer immunotherapy and associated immune-related adverse events. **(A)** Mechanisms of circRNA-mediated dysregulation of immune checkpoint (PD-1, CTLA-4, and PD-L1) inhibitors and their associations with organ-specific toxicity. **(B)** Potential circRNA -driven intervention strategies, including predictive biomarkers and circRNA vaccines, and side effects caused by immunogenicity.

While the study of circRNAs in immunotherapy-induced toxicity is still in its infancy, accumulating evidence indicates their potential involvement in regulating immune responses and inflammatory pathways. Future research may unlock their utility as both biomarkers and therapeutic modulators to minimize toxicity and improve the safety of immunotherapeutic regimens. To visually summarize these insights, Figure 4 schematically depicts the roles of circular RNAs (circRNAs) in cancer immunotherapy-induced toxicity.

### CircRNAs as biomarkers and therapeutic targets in oncotherapy-induced toxicity

Cancer therapy-induced toxicity is one of the most significant etiological factors underlying disease morbidity and mortality.^118^ CircRNAs have been studied as promising therapeutic molecules for early prevention, diagnosis, clinical intervention, and even predicting how well a disease will respond to therapy,^119^ owing to their inherent characteristics, such as high stability, long half-life, low immunogenicity, translatability, and tissue and developmental stage specificity.^120^^,^^121^ Numerous strategies have been devised to alter circRNA levels and target them for therapeutic purposes.^29^ To modulate circRNA levels, specific siRNAs or shRNAs that target back-splice junctions and CRISPR/Cas9-mediated editing techniques have also been employed.^122^ CircRNA-based therapies require efficacious delivery mechanisms because, once internalized, circRNAs tend to sequester inside endosomal compartments and are unable to traverse cell membranes, naturally.^123^ CircRNAs and other RNAs are most commonly transported via lipid nanoparticles (LNPs).^124^ Following endocytosis, LNPs cause the endosomal membrane to become unstable, allowing circRNAs to enter the cytoplasm. Additionally, circRNAs may be efficiently delivered and overexpressed *in vivo* via lentiviral and adenoviral vectors.^125^ For instance, circRNAs delivered by adeno-associated viruses (AAVs) improve cardiovascular function in transverse aortic constriction (TAC) animals.^126^ Doxorubicin-induced cardiotoxicity in mice was partially evaded by overexpressing conserved circITCH via the use of an AAV9 vector.^5^ RNA transport and its levels are regulated by means of exosomes derived from different sources. Exosomes shield RNAs from degradation and encourage their cellular absorption without inducing immunological reactions, thus opening new possibilities for *in vivo* research.^127^ Most interestingly, few circular RNAs with protein coding potential have been discovered. Translation of a designed circRNA with an IRES is possible *in vivo*.^128^ CircRNAs are more stable than linear RNAs in cells and human fluid because of their covalently closed ring structure, making them resistant to exonucleases.^129^ A circRNA with a prolonged half-life may continuously synthesize larger amounts of proteins.^128^ Hence, the future of circRNA-based therapies depends on the development of targeted and efficient strategies. Pertinent approaches for clinical therapies include the overexpression of circRNAs that impede disease progression or the silencing of carcinogenic circRNAs.^130^ Although the extensive formation of circRNAs limits this technique, druggable circRNAs that rely on *in vitro* or chemical synthesis are similarly fascinating molecules for drug discovery. Prospective research has focused on the safety and effectiveness of the nanoparticles and exosomes. Hence, the application of circRNA-based therapies would be encouraged by more realistic strategies that target or deliver circRNAs *in vivo*.^127^ Nevertheless, more innovative diagnostic techniques and improved treatment methods employing circRNAs as a mode of intervention are needed for clinical management.

### CircRNAs in the mechanisms of resistance to therapeutic toxicity

Resistance to treatment-induced toxicity is a complex phenomenon observed in both tumor and normal cells during cancer therapy. While such resistance can protect normal tissues, it may also reduce therapeutic efficacy by enabling tumor cells to evade drug-induced cell death.^131^ Emerging evidence suggests that circRNAs are involved in regulating cellular responses that contribute to this resistance, particularly in the context of chemotherapy and radiotherapy.^132^ CircRNAs influence various mechanisms underlying treatment resistance, including drug efflux, DNA damage repair, apoptosis inhibition, and oxidative stress regulation.^133^ For instance, some circRNAs act as ceRNAs by sponging microRNAs that would otherwise suppress drug resistance-related genes. An example is circRNA CDR1as, which sponges miR-7 and leads to the upregulation of anti-apoptotic or pro-survival pathways, contributing to chemoresistance in several cancers.^134^

Moreover, circRNAs can modulate DNA repair pathways, allowing tumor cells to withstand genotoxic stress from radiotherapy or certain chemotherapeutics. For example, circPVT1 has been shown to promote cisplatin resistance by enhancing the expression of DNA repair proteins and anti-apoptotic regulators.^135^ CircRNAs are also involved in the regulation of oxidative stress by controlling the expression of antioxidant genes. This helps both cancer and normal cells adapt to the ROS generated during therapy, reducing toxicity but also potentially enabling tumor survival.^136^

Importantly, these resistance mechanisms are not limited to tumor cells, as similar pathways may protect normal tissues, influencing the overall toxicity profile of treatment. However, the dual role of circRNAs as (i) protectors of normal cells and (ii) enablers of tumor resistance presents a therapeutic challenge. Understanding how circRNAs contribute to treatment-induced toxicity resistance could inform strategies to selectively sensitize tumor cells while preserving normal tissue function.

### Limitations and recommendations

Despite growing interest in circRNAs as diagnostic and prognostic biomarkers, several technical and analytical challenges hinder their clinical translation. Key among these are concerns related to specificity, sensitivity, and standardization in their detection.^137^ Specificity is a major concern owing to the high sequence similarity between circRNAs and their linear counterparts, making it difficult to distinguish circRNAs from linear RNAs via traditional qRT‒PCR or RNA-seq methods unless specific back-splice junction (BSJ)-spanning primers or probes are used. Improper primer design or insufficient depth of sequencing can result in false positive results or inaccurate quantification. Similarly, the sensitivity of circRNA detection is also limited by the generally low abundance of circRNAs in most tissues and body fluids, especially under normal physiological conditions. While circRNAs are relatively stable because of their covalently closed-loop structure, their detection in plasma, serum, or exosomes often requires enrichment steps such as RNase R treatment or highly sensitive amplification methods, which can introduce variability and reduce reproducibility.^36^ Moreover, the technical limitations in circRNA detection are compounded by the lack of standardized protocols and databases.^138^^,^^139^ Different computational tools and pipelines, such as CIRCexplorer, find_circ, and circRNA_finder, yield variable results, making cross-study comparisons difficult.^140^ Additionally, many reported circRNAs have yet to be functionally validated, raising concerns about the biological relevance of some candidates. To address these issues, improvements in bioinformatics pipelines, the development of standardized assays such as digital PCR, circRNA arrays, and large-scale validation studies are urgently needed. Overcoming these technical barriers is essential for the successful clinical integration of circRNAs as reliable biomarkers in cancer and other diseases.

Although appreciable progress has been made in identifying and characterizing circRNAs, more studies are needed to address important unknown aspects and constraints in uncovering and recognizing the biological activities of circRNAs.^141^ CircRNA activities are highly context dependent, and in many tissues and disorders, they impart their functions by regulating one or more molecular pathways. However, substantial research has focused on the functions of circRNAs as competing endogenous RNAs in illnesses such as cancer; however, the reported number of circRNAs that can enhance disease progression through miRNA sponges is far less than what was previously assumed. There was no positive correlation between the ability of circRNAs to sponge miRNAs and their miRNA binding sites. There can be a circRNA with a single miRNA binding site that can still be functional. Since most physiological changes in circRNAs do not affect miRNA activity, the ratio of circRNA/miRNA and the relationship between the circRNA and miRNA binding sites and the miRNAs and mRNA target sites may be crucial for suppressing the target through the ceRNA mechanism. This may be attributed to the fact that many circRNAs are expressed at low levels. Understanding how circRNAs function as miRNA sponges requires precise measurements of their copy numbers and circRNA/miRNA ratios in both healthy and diseased cells and tissues. A well-documented functional mechanism by which circRNAs are involved in the toxicity generated by oncotherapy is the “circRNA‒protein interaction”, which involves one circRNA. Certain disease conditions may cause RNA to bind primarily to one protein, a complex of proteins, or many circRNAs may create a circRNA‒protein complex. Nevertheless, circRNAs appear to have a lower RBP binding density than their linear counterparts do, necessitating the development of innovative and effective techniques for evaluating possible circRNA binding. Furthermore, circRNAs appear to have several modes of activity in various illnesses, indicating that their effect on a particular disease phenotype is presumably context dependent. To confirm circRNA functions and disregard false positive effects, appropriate controls and rescue tests are still essential. Similar research methodologies can be extended to broader domains, such as cancer therapy-induced toxicity, as advancements in the study of circRNA functions have largely been made with respect to either tumor suppression or development.

## Conclusion and perspective

In the last decade, various studies have shed light on the functional mechanisms of circRNAs, which range from miRNA sponging and transcriptional regulation to binding proteins at tertiary structures and protein translation. Nonetheless, the functional intricacies of circRNAs in various forms of tumors are becoming increasingly complex. Although the number of people diagnosed with cancer has increased in recent years, the number of cancer survivors has increased dramatically owing to technological advancements in the field of medicine, and the problem of toxicity from anticancer therapies has ensued. In this context, it is essential to integrate the relationship and mechanistic interplay between circRNAs and the toxicity of various systems and organs produced during the course of anticancer therapy. To date, numerous studies have explored the role of ncRNAs in regulating chemotherapy-induced toxicity, and significant progress has been made in the field. However, various challenges and limitations remain regarding their clinical usability. In terms of basic research, the understanding of circRNAs remains relatively limited compared with that oflncRNAs and miRNAs.

Concerning disease diagnosis, the potential of circRNAs to serve as diagnostic biomarkers cannot be undermined; nevertheless, the specificity and sensitivity of circRNAs in most studies have not been adequately evaluated. Moreover, the expression level of circRNAs is generally low, which makes clinical detection more challenging. With respect to treatment, it is also crucial for circRNAs to move from the laboratory to the clinic, as most circRNAs employ miRNA sponging as their putative mechanism of action; however, achieving significant therapeutic effects through miRNA sponges is rather difficult, as the stability of miRNA sponges is largely unknown. These issues must be addressed through further research.

In summary, in addition to the extensive original research on the regulatory roles of circRNAs in tumorigenesis and development, studies on the mechanisms of circRNA-mediated modulation of oncotherapy-induced toxicity during cancer treatment remain limited. More studies in this research sphere will not only contribute to a more in-depth understanding of the molecular mechanism of circRNAs in tumors but also provide a new method and strategy for monitoring, preventing, and treating a variety of adverse reactions caused by anticancer therapy, which provides new hope for anticancer therapy for affected patients. Overall, circRNAs have emerged as key regulatory molecules influencing multiple cellular processes associated with cancer treatment-induced toxicity. Their involvement in oxidative stress, apoptosis, and homeostatic imbalances highlights their potential as both biomarkers and therapeutic targets. Nevertheless, further research is essential to fully harness their clinical utility in mitigating treatment-related adverse effects in cancer.

## CRediT authorship contribution statement

**Jiawen Xian:** Writing – original draft, Visualization. **Javeria Qadir:** Writing – original draft, Visualization. **Burton B. Yang:** Writing – review & editing. **Ting Ye:** Writing – review & editing, Supervision, Resources, Funding acquisition.

## Funding

This work was supported by the National Natural Science Youth Fund, China (No. 82003138), the Cooperative Scientific Research Project of the "Chunhui Plan" of the Ministry of Education, China (No. HZKY20220575), the Haiju plan High-End Talent Introduction Program of the Sichuan Provincial Department of Science and Technology (China) (No. 2025HJRC0036), and the Medical Science and Technology Development Project of Clinical Medicine at Southwest Medical University (China) (No. 2024LCYXZX24).

## Conflict of interests

The authors declare that they have no competing interests.
